# The Human Organ Atlas

**DOI:** 10.1126/sciadv.adz2240

**Published:** 2026-03-11

**Authors:** Claire L. Walsh, Joseph Brunet, David Stansby, Guillaume Gaisné, Yang Zhou, Maximilian Ackermann, Alexandre Bellier, Camille Berruyer, Axel Bocciarelli, Marjolaine Bodin, Bernadette S. de Bakker, Hector Dejea, Alejandro De Maria Antolinos, Klaus Engel, Andy Götz, Joseph Jacob, Daniel Jonigk, Joanna Purzycka, Theresa Urban, Stijn E. Verleden, Ruikang Xue, Paul Tafforeau, Peter D. Lee

**Affiliations:** ^1^ Department of Mechanical Engineering, University College London, London, UK.; ^2^ ESRF, The European Synchrotron, 71 Avenue des Martyrs, CS40220, 38043 Grenoble Cedex 9, France.; ^3^ Advanced Research Computing Centre, University College London, London, UK.; ^4^ Institute of Functional and Clinical Anatomy, University Medical Center of the Johannes Gutenberg-University Mainz, Mainz, Germany.; ^5^ Department of Anatomy (LADAF), Univ. Grenoble Alpes, CIC INSERM 1406, Grenoble, France.; ^6^ Department of Obstetrics and Gynecology, Amsterdam UMC location University of Amsterdam, Meibergdreef 9, Amsterdam, Netherlands.; ^7^ Amsterdam Reproduction and Development research institute, Amsterdam, Netherlands.; ^8^ Siemens Healthineers AG, Erlangen 91052, Germany.; ^9^ UCL Respiratory, University College London, London, UK.; ^10^ Satsuma Lab, Hawkes Institute, University College London, London, UK.; ^11^ Institute of Pathology, University Medical Center RWTH University of Aachen, Aachen, Germany.; ^12^ Biomedical Research in Endstage and Obstructive Lung Disease Hannover (BREATH), German Center for Lung Research (DZL), Hannover, Germany.; ^13^ Antwerp Surgical Training, Anatomy and Research Centre (ASTARC), University of Antwerp, Wilrijk, Belgium.

## Abstract

We present the Human Organ Atlas (HOA), an open data repository making accessible multiscale three-dimensional imaging of human organs. The repository also provides software tools and training resources enabling worldwide access, sharing, and analysis of these datasets, facilitating further research and the continued expansion of the HOA. The images are generated using a synchrotron imaging technique, hierarchical phase-contrast tomography (HiP-CT), that uses the ESRF’s Extremely Brilliant Source, spanning whole-organ imaging at ~20 micrometers/voxel with local volumes of interest within intact organs imaged down to ~1 micrometer/voxel. This offers a comprehensive exploration of human anatomy, providing unparalleled insights into intricate structures and spatial relationships. The HOA offers researchers, clinicians, and educators a valuable resource for anatomical study, image analysis, medical education, and large-scale data mining.

## Teaser

The Human Organ Atlas provides open access to 3D images of human anatomy spanning whole organs to cellular scale resolution.

## INTRODUCTION

Human organs are intricate three-dimensional (3D) structures. Their hierarchical organization, from the extracellular matrix to cells to functional units to tissues to organs, underpins functional properties, allowing both coordination and specialization. Mapping the 3D morphology and spatial distribution of biological structures at these multiple scales can provide substantial healthcare and biological impacts including bounding what constitutes healthy and pathological variability of structures, linking between morphology and transcriptome, and providing data to explicitly simulate function from structure.

Efforts to map the entire human body at the cellular level and the tissue level have been ongoing for some time, with 3D imaging as a cornerstone in many of these efforts (*1*–*7*). In recent years, we have developed the hierarchical phase-contrast tomography (HiP-CT) technique, which uses synchrotron x-ray tomography and creates hierarchical image volumes of ex vivo intact human organs, spanning the scales from the single cell to whole organ (*8*, *9*). HiP-CT imaging of a human organ has typically involved imaging at multiple resolutions: initial imaging of the whole sample with an overview scan at an isotropic voxel size of ~20 μm, followed by zoom scans in selected volumes of interest at a configurable isotropic voxel size of ~1 to 6 μm, all within the intact organ. The current lowest achieved voxel size is 0.65 μm. The images at each resolution can be rigidly aligned to one another creating hierarchical datasets that provide histological level detail in 3D at user selected locations within whole organs without physical sectioning. With technical developments since the original HiP-CT results, the speed and consistency of imaging have markedly increased, allowing for routine and rapid (2 to 8 hours depending on organ size and scan parameters; see fig. S1) multiresolution scanning of human organs, which has generated a large database of 3D hierarchical organ images.

**Fig. 1.**
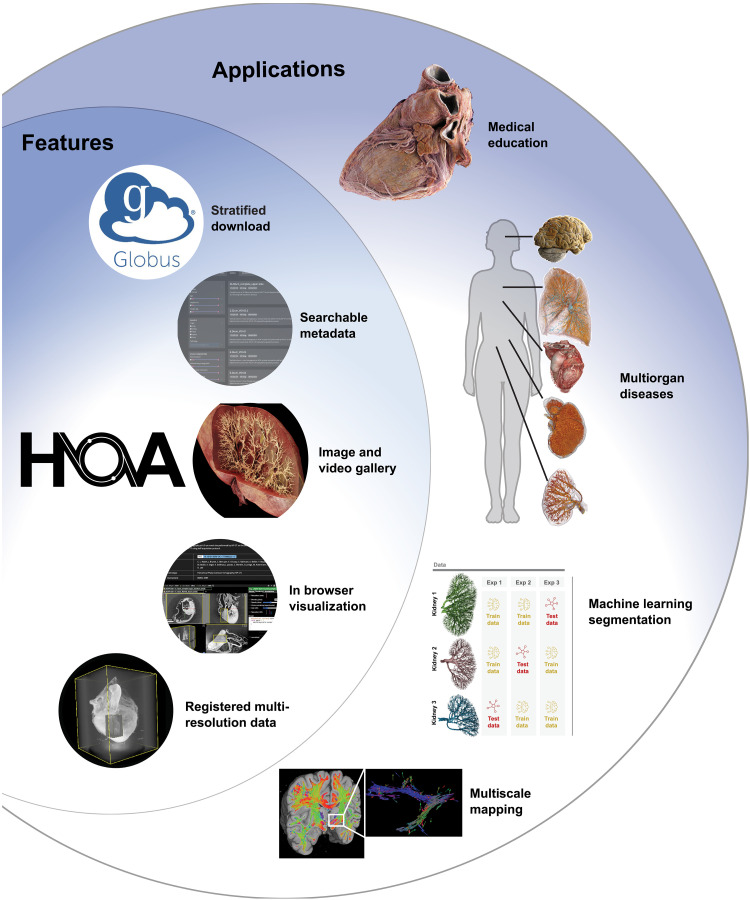
Overview of the HOA. Features include open data download, search tools based on metadata, image/video galleries, browser-based visualization, and registered hierarchical datasets. “Applications” show some of possible uses that the HOA has and will enable for researchers around the world, including anatomical visualization and training; studying multisystemic disease, e.g., COVID-19 and hypertension; machine learning image segmentation challenges [panel adapted from (*30*), reproduced under a Creative Commons Attribution 4.0 International License (https://creativecommons.org/licenses/by/4.0/)]; and mapping of texture features such as white matter orientation in the brain.

HiP-CT has already shown impact in biomedical fields (*10*–*16*), but the technique requires the small x-ray source size, high energy, large beam size, and long propagation distances available on beamlines BM05 and BM18 at the European Synchrotron Radiation Facility (ESRF) (*17*). This limits accessibility of the technique for those without the resource or training to independently apply it. To maximize the utility and impact of HiP-CT, we have developed a data portal, the Human Organ Atlas (HOA), to ensure that data that we collect is findable, accessible, interoperable, and reusable (FAIR) (Fig. 1A) (*18*).

Here, we outline the features of the HOA (Fig. 1), the organization of the data portal, and the data now available. We then provide demonstrators for how open data in the HOA may be used by different communities through two examples: (i) application to machine learning segmentation using multiresolution images and (ii) application to anatomical training and education through advanced visualization techniques. We lastly discuss the future direction for development of the HOA.

## RESULTS

### Features and organization of the HOA

**Fig. 2.**
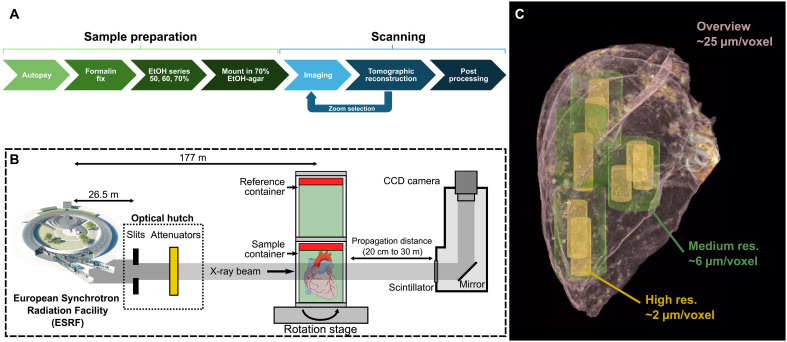
Overview of the HiP-CT method used to acquire data for the HOA. (**A**) Overview of the HiP-CT method pipeline [adapted from (*8*) under a Creative Commons Attribution 4.0 International License (https://creativecommons.org/licenses/by/4.0/)]. (**B**) Diagram showing the key components of the imaging setup at ESRF BM18 [adapted from (*10*) under a Creative Commons Attribution 4.0 International License (https://creativecommons.org/licenses/by/4.0/)]. (**C**) Example of the hierarchically aligned volumes of image data from HiP-CT scan of the lung of S-20-29. In each case, there is an overview scan and then various higher-resolution scans aligned within it. In this example, there are medium-resolution and high-resolution scans at 6 and 2 μm/voxel, respectively. Multiple medium- and high-resolution scans can be made within a single sample, and all can be aligned to the overview scan.

The HOA data portal is available at human-organ-atlas.esrf.eu. All datasets are acquired using the HiP-CT method (Fig. 2, A and B) performed on ex vivo human organs, following methods and protocols that have been previously described (*8*, *9*). The resulting 3D hierarchical datasets provide detailed organ overviews with multiple higher-resolution zoom datasets nested within (Fig. 2C).

**Fig. 3.**
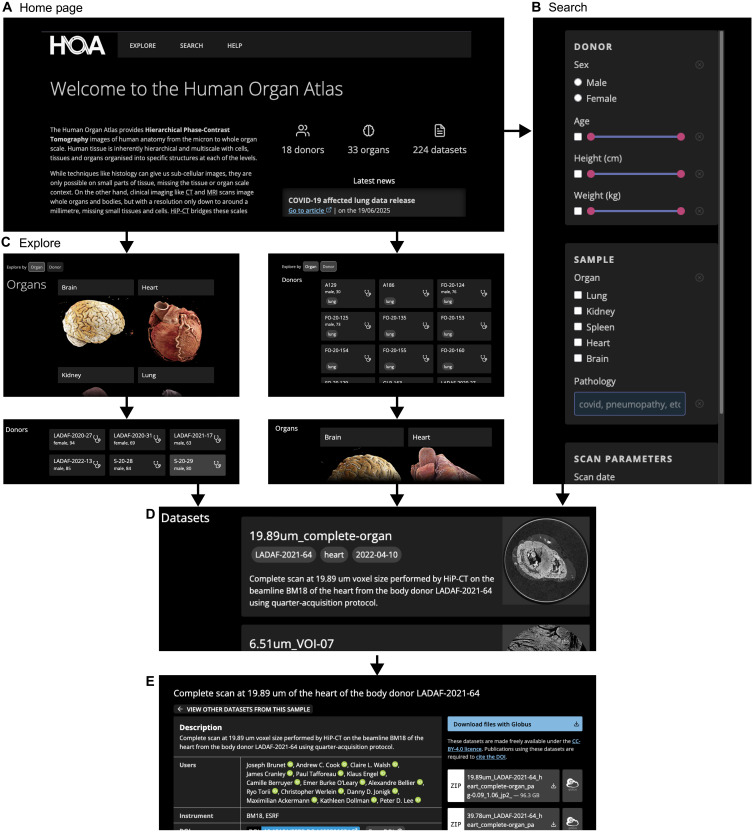
Overview of the HOA portal. (**A**) The HOA home page with tabs for different pages listed across the top. To discover datasets, users can either go to (**B**) the Search tab to search based on medical, scan, and demographic metadata or go to (**C**) the Explore tab. Within the Explore tab (C) datasets can be ordered first by organ [(C), left column] or by donor [(C), right column]. From both Search and Explore tabs, the user is then presented with (**D**) a list of datasets and, when one dataset is chosen, is taken to (**E**) the dataset page with in-browser visualization and download options for the various downsampled datasets and metadata.

The HOA portal (Fig. 3A) has features to make the data FAIR for a broad variety of users. The data are made available under a CC-BY-4.0 license which requires users of the data to cite the DOIs of the datasets that they reuse.

To make the data findable, the “Explore” tab allows data to be viewed either by Donor > Organ > Dataset or by Organ > Donor > Dataset (Fig. 3C), while the “Search” tab (Fig. 3B) provides a graphical interface to search and filter through the available metadata for each dataset.

To make the data accessible, each dataset page (Fig. 3E) provides data download via the Globus data transfer system. Data are provided and stored in the JPEG2000 image format (*19*) with a compression factor of 10 to enable faster download and more efficient storage. Multiple downsampled versions of the same dataset are available for download (Fig. 3E) with each dataset’s highest downsampling level having a compressed file size of 400 MB or less, making lower-resolution versions of the data accessible to those with limited computational resources.

To make the data interoperable, metadata for each dataset are available for download as a JSON file that conforms to a strict schema (*20*). These metadata fall under six broad categories: information about the data itself, scanning parameters, donor information, sample preparation, image registration transforms, and attribution data. A copy of the metadata for all datasets is also hosted alongside allowing for programmatic interaction with the metadata. Although best efforts are made to collect and attach as much medical history metadata as possible, in general, it cannot be taken as complete for each donor due to parts of the medical history collection pipeline outside of the projects control. All the above features enable reusability across multiple scientific and education fields, e.g., medical, bioimage analysts, medical illustrators, educators, and image processing.

For each dataset, a web-based visualization tool, neuroglancer (Fig. 3E) (*21*), allows users anywhere in the world quickly view and interact with any dataset. With just a web browser and no additional software, users can assess scan quality, identify features of interest, locate zooms within each overview scan, and add minimal shareable annotations before deciding whether to download the datasets for local analysis.

The higher-resolution zoom datasets are registered to the appropriate whole-organ overview dataset, and the transforms for these registrations are provided in the dataset metadata. This powers visualization of the zoom scans in the context of overview scans within neuroglancer on the website.

A tutorial and help page provide instruction on how to navigate and use the portal, as well as basic tutorials on how to download the datasets, use neuroglancer, and load and analyze data in Fiji (*22*). The hoa-tools Python package (*23*) provides more advanced tools for data operations and processing through the scientific Python ecosystem, making use of xarray, dask, and numpy (*24*, *25*).

### Current data in the HOA

At present, the HOA contains 298 3D images from 24 individual donors across 10 organs: brain, colon, heart, kidney, liver, lung, prostate, spleen, testis, and uterus (Table 1). Donors were initially sourced from two large European biobanks: the Hannover Unified Biobank and the Laboratoire d’Anatomie Des Alpes Françaises (LADAF). Given the nature of body donation to such programs, at the time of writing, the HOA is more representative of older individuals (mean age, 73 years; range, 30 to 94 years) and male donors, with 13 males, 5 females, and 4 donors of unknown sex. Over time, the number of biobanks feeding HOA has diversified past the initial two to more than a dozen, which will help improve the diversity of donors with future data releases. To facilitate cross-organ analysis and enable studies of multiorgan anatomical relationships, a concerted effort has been made to acquire and curate datasets for multiple organs from the same donor, notably LADAF-2021-17, for whom datasets are available for the brain, colon, heart, kidney, liver, lung, prostate, spleen, and testis (Table 1).

**Table 1.** Overview of datasets available in the HOA at the time of writing. Organ columns on the right denote the number of overview datasets plus the number of zoom datasets for each organ/donor.

Donor ID	Sex	Age	Brain	Colon	Heart	Kidney	Liver	Lung	Prostate	Spleen	Testis	Uterus
A129	M	30						0 + 1				
A186								1 + 1				
AUMC-005												1 + 0
FO-20-124	M	76						1 + 10				
FO-20-125	M	73						1 + 0				
FO-20-129	M	54						2 + 20				
FO-20-135								1 + 0				
FO-20-153								1 + 0				
FO-20-154								1 + 9				
FO-20-155								1 + 8				
FO-20-160								1 + 0				
GLR-163	M	77						0 + 1				
LADAF-2020-27	F	94			2 + 5	2 + 2	2 + 0	2 + 23		1 + 2		
LADAF-2020-31	F	69	1 + 5		2 + 10	1 + 1						
LADAF-2021-17	M	63	2 + 18	1 + 4	2 + 18	2 + 10	1 + 4	2 + 10	1 + 0	1 + 0	2 + 2	
LADAF-2021-64	F	87			1 + 14							
LADAF-2022-13	M	85				2 + 0			1 + 0			
LADAF-2022-16	M	75							1 + 0	1 + 0		
LADAF-2022-27	M	85				2 + 0						
S-20-28	M	84			2 + 8	1 + 9		1 + 0				
S-20-29	M	80	2 + 18		2 + 14	1 + 4		1 + 7				
S-21-33	M	72	1 + 0									
S-21-46	F	68	1 + 0									
S-22-16	M	73	1 + 0									

**Fig. 4.**
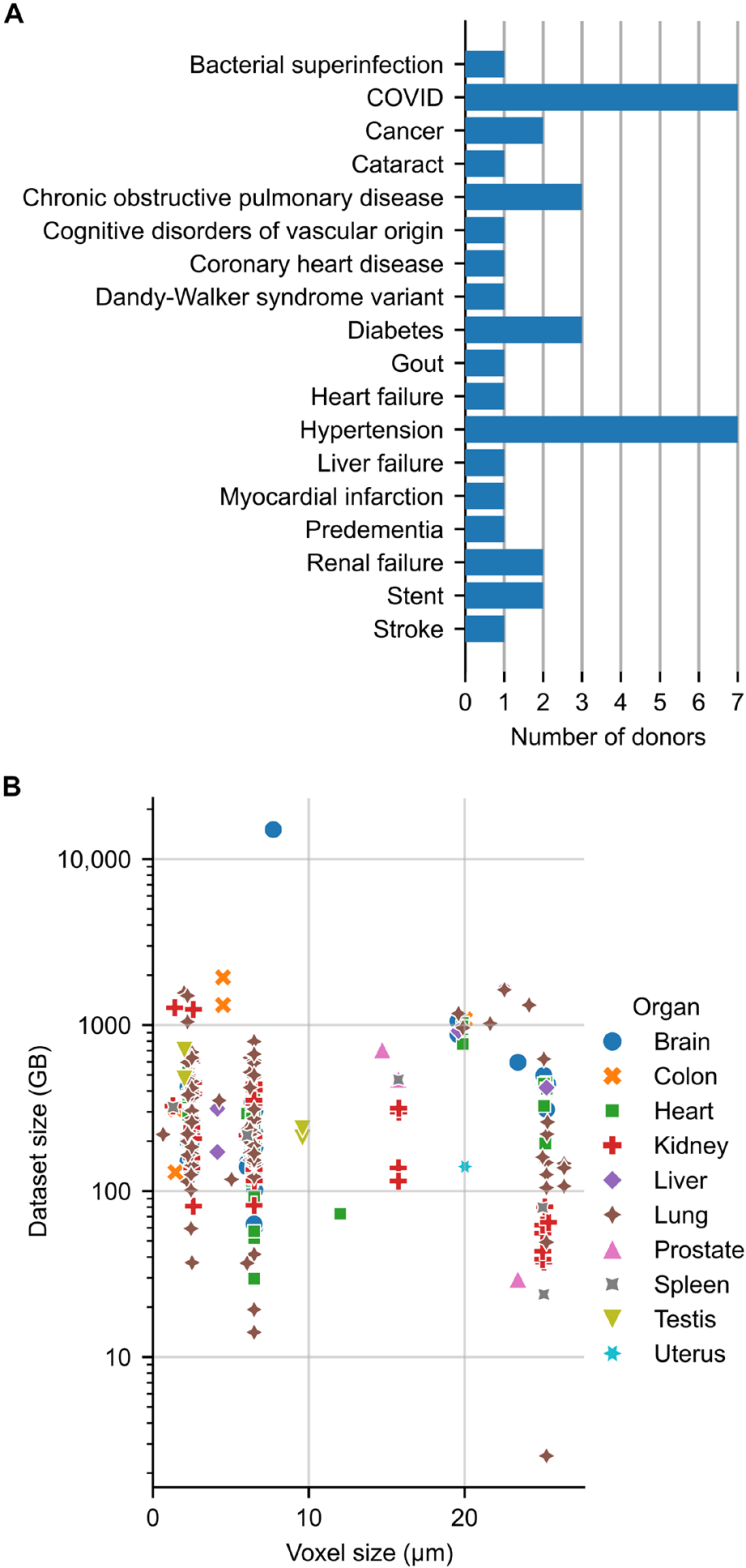
Overview of data now in the HOA. (**A**) An overview of key medical conditions represented in the atlas. These medical conditions are provided as metadata and allow researchers with an interest in a specific disease to find and use data that are relevant to their research questions. Note the higher proportion of age-related diseases such as cancer and hypertension as well as recently widespread diseases, e.g., COVID-19, and some rarer diseases such as Dandy-Walker syndrome. (**B**) The voxel size of each dataset against dataset size, with organs separated by marker style and color. Many datasets are more than 200 GB, and some are more than 1 TB, highlighting the importance of having in-browser viewing and downsampled datasets available for download.

Within available datasets, there are a large variety of total data sizes (summarized in Fig. 4A). Seventeen (6%) are 1 TB or greater, 44 (15%) are more than 500 GB, and 265 (90%) are more than 100 GB. Dataset size does not correlate with the type of dataset (overview or zoom) and is typically in the range 30 GB to 1 TB.

Given the ex vivo nature of HiP-CT and the age of donors, the HOA contains a wide variety of age-related pathologies, such as hypertension and cancer, as well as a high proportion of COVID-19 cases, reflecting both the global causes of death in recent years (*26*) and the roots of the HiP-CT method that was developed to better understand the changes to different organs due to COVID-19 during the pandemic. There are also rare pathologies within the HOA, e.g., in donor S-20-29 Dandy-Walker syndrome, a congenital malformation of the cerebellum that affects less than 1 in 30,000 individuals (Fig. 4B) (*27*, *28*). Together, these samples provide a rich imaging database to study diseases that are leading causes of death in the global north today, as well as rare pathologies that few researchers may have direct access to.

### Applications in machine learning for quantification of structures

A key application of the HOA is the provision of a curated 3D hierarchical imaging dataset for the biomedical machine learning community, offering several distinct possibilities for researchers across supervised, semisupervised, and unsupervised regimes. For example, researchers can (i) leverage the 3D isotropic nature of the data at any one resolution to segment 3D structures, (ii) make use of the alignment of the hierarchical datasets for super-resolution applications, or (iii) make use of the large quantity of curated 3D data to train self-supervised foundation models.

For supervised approaches, researchers can download datasets freely and perform their own manual segmentation of biological structures. These segmentations can then be used both directly for quantification (*16*, *29*) or to create training datasets for supervised machine learning approaches (*30*, *31*). Models and methods developed should be directly applicable to data acquired with other phase-contrast–based imaging methods [e.g., (*32*, *33*)]. The addition of style transfer methods to transform imaging from one modality to another also opens the possibility of training on HOA data but applying methods to other modalities like absorption-based computed tomography (CT) or magnetic resonance imaging (MRI).

In addition to supervised methods, the HOA provides a large, curated 3D data pool for training unsupervised models or foundation models using, e.g., masked autoencoders (*34*). The limited availability of open-source large-scale 3D datasets has been increasingly highlighted as a limitation for the development of 3D foundation models (*35*, *36*). Given the growing significance of these models including notable models such as the segment anything model (SAM) and MedSAM (*37*–*39*) and due to their capacity to provide zero-shot or one-shot segmentations (*40*), we anticipate this to be a large use case for HOA datasets.

**Fig. 5.**
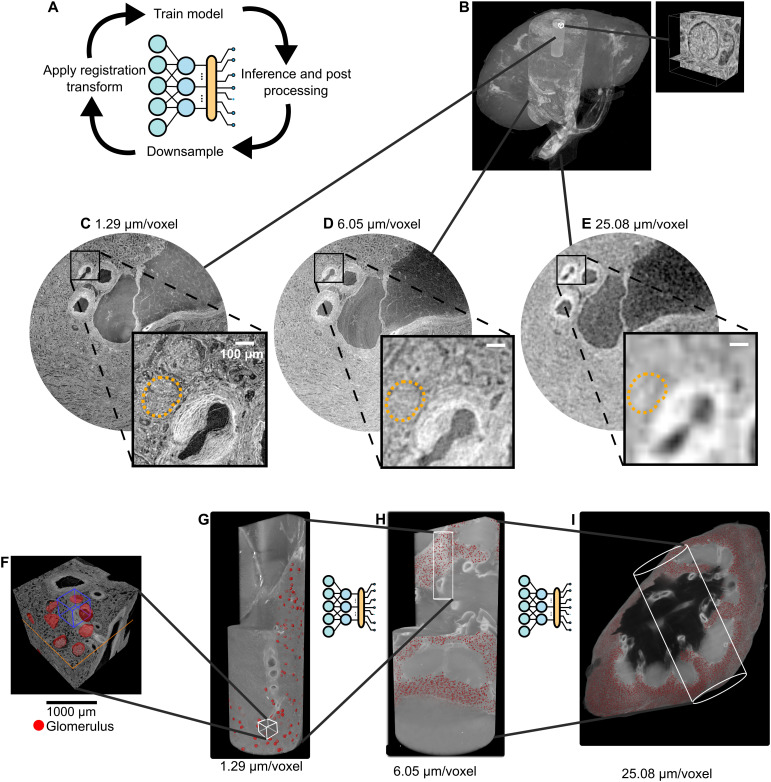
Using registered hierarchical datasets from the HOA to perform a super-resolved segmentation. (**A**) Overview of the approach. (**B**) Hierarchical dataset for human left kidney LADAF-2020-27, with renal glomerulus images at 1.29 μm. (**C** to **E**) The glomeruli outline at each imaging resolution (orange dashed line). Note how the glomeruli cannot be reliably distinguished at 25 μm/voxel. (**F** to **I**) The manual annotations, predictions, and inference across the resolutions with the final predicted glomeruli distribution across the whole kidney. Panel adapted from results in (*44*) under a Creative Commons Attribution Non-Commercial 4.0 International License (https://creativecommons.org/licenses/by/4.0/).

A unique feature of HOA datasets that can be used by researchers is the hierarchical nature, i.e., aligned data of the same structures imaged at multiple resolutions. These datasets are of particular interest for super-resolution applications or machine learning–based image compression (*41*, *42*). Another application is super-resolution segmentation, i.e., propagating segmentations made on higher-resolution image volumes to lower-resolution image volumes within the same organ as pseudolabels (Fig. 5A). This can allow small subresolution structures to be mapped to whole-organ imaging, enabling spatial distribution or morphology of these structures to be profiled across never examined length scales.

As an example, in the human kidney, the tissue functional unit [“the smallest tissue organization that performs a unique physiologic function and is replicated multiple times in a whole organ”; (*43*)] is the renal glomerulus, a ball-shaped tuft of capillaries ~100 to 300 μm in diameter (Fig. 5B). In the kidney data available for donor LADAF-2020-27, Glomeruli can be easily segmented with very high accuracy in the highest-resolution datasets (1.29 μm/voxel) (Fig. 5C), at meso-resolution (~6 μm/voxel) glomeruli can be seen, but accurate segmentation of the boundary can be challenging (Fig. 5D), and, at the lowest-resolution overview of this organ (25 μm/voxel), only very prominent glomeruli can be precisely resolved (Fig. 5E).

Figure 5F shows the output of a multiscale segmentation pipeline applied to this kidney dataset (*44*), demonstrating the effectiveness of using the nn-UNet model (*45*) as the benchmark to propagate glomeruli segmentation to the scale of the whole kidney. Such a concept is applicable to other functional units across different organs (*46*).

### Applications to anatomical visualization and medical education

Datasets in the HOA surpass clinical CT or ex vivo MRI (*47*) voxel size by one to two orders of magnitude and, unlike whole-organ light sheet microscopy (*48*), offer isotropic voxel shape. Our voxel size is comparable to state-of-the-art whole-organ serial-section histology (*7*), but, unlike histology, carrying out the imaging using HiP-CT is nondestructive, maintaining anatomical integrity and allowing imaging all the way from the organ to the cellular scale.

**Fig. 6.**
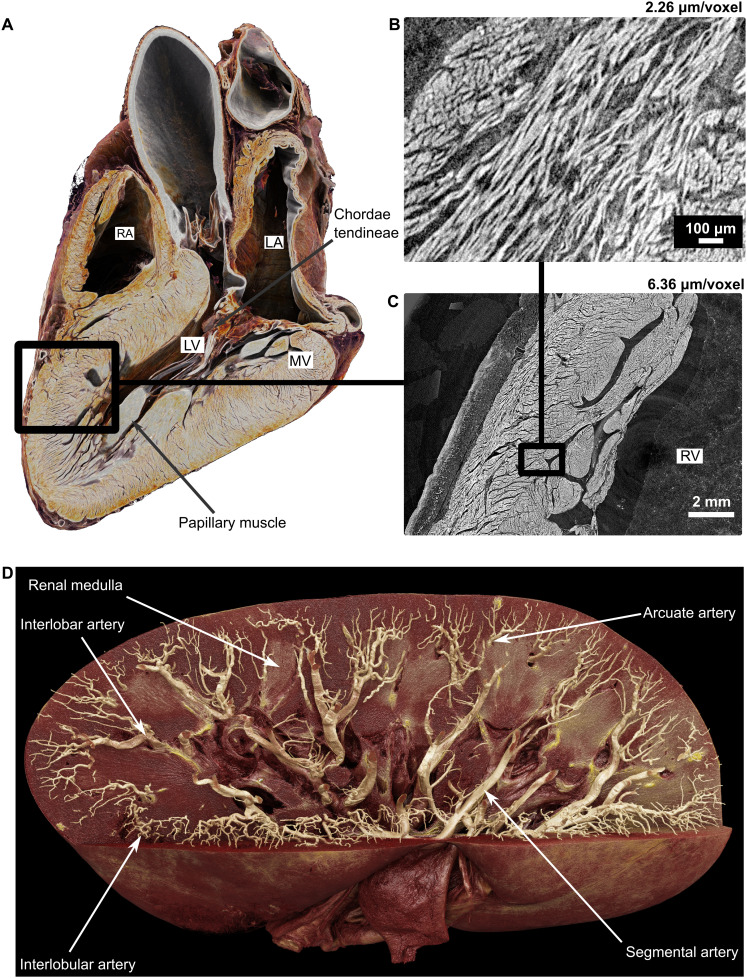
Application of HOA data to creating anatomical teaching materials. A full human heart imaged at 19.85 μm/voxel, allowing detailed visualization of the ventricular myocardium within the intact organ. (**A**) A virtual longitudinal section with anatomical landmarks labeled, including the left (LV) and right (RV) ventricles, mitral valve (MV), right atrium (RA), left atrium (LA), papillary muscle, and chordae tendineae. (**B** and **C**) Hierarchical zoom-ins of the left ventricular wall, imaged at 6.36 and 2.26 μm/voxel, respectively, revealing the detailed myocardial microstructure such as cardiomyocyte fibers. (**D**) An intact human kidney imaged at 25 μm/voxel, where the arterial tree has been segmented before rendering. Each anatomically defined vessel is shown in a real example from the renal artery of ~2 cm in diameter down to the interlobular arterioles of ~50-μm radius.

The multiscale nature and increasing number of donors and organs available make the HOA a valuable tool for anatomy education and research. Visualizing detailed 3D structures, such as heart valves (Fig. 6A) and kidney vasculature (Fig. 6D), is possible at multiple scales. Unlike idealized anatomical models, real patient image data offer a more nuanced understanding of human anatomy. The application of HiP-CT for anatomy in a research context in health and disease has been demonstrated through application to the cardiac conduction system (*10*), hippocampal arteries of the brain (*15*), and 3D structure of congenital airway malformation (*14*). In the medical education field, 3D modelling can be used to transfer schematic anatomical knowledge to real anatomy applicable in clinical or surgical practice.

Combining HOA data with advanced visualization and rendering techniques, such as 3D Gaussian splatting (*49*), has demonstrated the feasibility of interacting with 3D rendered HOA data, e.g., manipulating, rotating, and zooming in on specific structures in real time. 3D Gaussian splatting considerably reduces the size of the data while maintaining high quality. This interactivity facilitates active learning, helping students understand spatial relationships between complex 3D anatomical structures, meaning that the HOA will have a central role in the next generation of anatomy education applications.

## DISCUSSION

The HOA is a highly unique resource, serving many multiresolution image datasets across multiple donors, organs, and organ types and providing metadata, online tools for visualization, and tutorials for the download and use of the datasets. This offers a comprehensive exploration of human anatomy, providing insights into the intricate structure and spatial relationships, from the whole organ to organ functional units and even down to some cellular structures. Making the data publicly available is already paying dividends, with studies outside our immediate team already making use of it (*50*–*54*).

As with any data, there are limitations and drawbacks that scientists should be aware of when using data from HOA. Due to the ex vivo nature of the imaging technique, shrinkage due to organ preparation and the collapse of large chambers (particularly visible in the left atrium of the heart in Fig. 6A) are present in most datasets. Blood vessels are also often collapsed, which poses challenges for annotating vasculature (*30*). Imaging at such high resolutions introduces the potential for radiation damage to the samples, but the HiP-CT acquisition protocol has been specifically tailored to prevent this (*55*).

For several donors, multiple organs are available in the portal (Table 1), providing an opportunity for the study of multisystemic diseases. For example, donor LADAF-2020-27 who had a history of hypertension has lung, heart, kidney, and spleen images available. Studying the impact of different diseases across the whole body is a primary avenue of research that the HOA will enable in the coming years.

The HOA continues to progress and evolve with more datasets, tutorials, and tools, and increasing data quality linked to the technical developments on the BM05 and BM18 beamlines at ESRF. Most data released, so far, were acquired at BM05 in the period before 2023. Since the BM18 beamline started operations in 2022, it has enabled increased propagation distances and larger beam sizes, increasing the speed and contrast sensitivity of scans (*17*). The HOA project is still actively collecting data and releasing these datasets to expand the diversity, quality, and size of data available.

A consortium has recently been formed at the ESRF around the HOA and HiP-CT imaging technique, the Human Organ Atlas Hub (HOAHub; https://mecheng.ucl.ac.uk/HOAHub). The HOAHub continues to collect data and to improve upon automated analysis methods, multimodal pipelines, sample curation for specific biomedical questions, as well as dynamic image–based modelling. Through the efforts of the consortium, data will continue to be released over the coming years. Alongside the imaging datasets, as part of further scientific studies, we plan to start providing derived datasets that have and can be used in further analysis, for example, manual and automated anatomical segmentations. For some samples, complementary datasets acquired using other techniques are also planned be provided in the future, including MRI, histology, and spatial transcriptomics.

We also plan to expand the tutorials and tools through linking to tools for simple data handling, more advanced image registration, and segmentation models, which will be provided as they are developed. Last, we plan to make continuous improvements to the metadata, which include making it interoperable with community adopted metadata ontologies [e.g., Uberon (*56*)].

The HOA offers researchers, clinicians, and educators a valuable resource for anatomical study, image analysis, medical education, and large-scale data mining through machine learning. With its user-friendly interface, searchable database and ever-growing number of datasets, the HOA provides FAIR data access to a unique imaging technique. By opening the data and technique up to a wider audience, we aim for scientists across various disciplines to advance understanding, diagnosis, and treatment in the realm of human health and medicine.

## MATERIALS AND METHODS

### Sample preparation and scanning

All organs were prepared following the protocols described in (*9*). Informed consent was obtained from all donors. All the scans were performed on the beamlines BM05 and BM18 of the ESRF (Grenoble, France) following the protocols of (*8*). Various configurations of optics and detectors were used and are provided for each dataset in their metadata.

### Data reconstruction and formatting

The tomographic reconstruction process followed is detailed in (*9*). After tomographic reconstruction, the 3D volumes are converted to JPEG2000 format. A lossy compression factor reduces the sizes of stored data by a factor of 10. The full-resolution data are progressively downsampled by power of 2 into lower-resolution datasets to provide smaller file size options for download. This ensures that the lowest-resolution dataset can fit into a wider range of hardware available to researchers. The 3D volumes are then ingested to the HOA portal via ESRF servers. All datasets are also converted to the chunked N5 (*57*) (older datasets) or OME-Zarr (*58*) (newer datasets) data format and stored online as a source for the browser-based neuroglancer visualization tool (*21*).

### Dataset registration

Each zoom dataset is registered to a corresponding respective overview dataset, using software available at (*59*). A common point in both datasets is manually selected to initialize the transform between the two datasets. A 128 × 128 × 32 voxel subvolume centered on this common point is taken from the overview dataset, and the corresponding subvolume in physical space taken from the zoom dataset. Because samples are fixed within their jars and do not move between overview and zoom imaging, a rigid transform is used during registration with five parameters: the isotropic voxel scale, three translation values, and rotation around the *z* axis. Each registration undergoes visual quality control to ensure that the same structures are colocated in the registered zoom and overview image.

This registration method is designed to provide a quick and reasonably accurate registration, allowing similar structures to be easily and qualitatively located across zoom and overview datasets. It also provides a starting transform for users who need single-voxel accurate registrations to run their own more precise registrations. The resulting transforms for each zoom dataset are provided in their metadata file.

### Metadata collection

Metadata collation is done through a mixture of manual process and automated pipelines. Collection of donor demographic and medical data is done in accordance with ethics guidelines of the respective biobanks, and sample preparation metadata are manually recorded. Scanning and reconstruction/image volume metadata are automatically collected during the reconstruction pipeline. These metadata are collated into a single structure and validated for consistency against a schema (*20*).

### Website technology

The portal is developed using Node.js and pnpm for package management. It is written using the JavaScript library React and TypeScript for maintainability and improvements over time. Vite is used to bundle the website, and it is served by an Nginx machine, deployed using Docker, on the ESRF’s servers.

The data curation and archiving within the portal leverages the DRAC (Data Repository for Advancing sCience) ecosystem (*60*), specifically designed to comply with the ESRF’s data policy (*61*). This policy aligns with Open Science and FAIR data principles, allowing users to publish results derived from data collected at the ESRF with unique DOIs. It guarantees that metadata are archived forever and aims to retain data for at least 10 years with the option of longer periods for high-value datasets. The ESRF repository is CoreTrustSeal certified (*62*), underlining its adherence to internationally recognized standards for reliable and sustainable data curation. DRAC integrates key components like ICAT (*63*) and ICAT+ for metadata management, sample tracking, and experiment logging, ensuring that all research data follow a structured life cycle from acquisition to long-term preservation.
