# Supplementary material for: The Human Organ Atlas
Source: Sci Adv. 2026 Mar 11;12(11):eadz2240. doi: 10.1126/sciadv.adz2240 (PMC12978218; doi:10.1126/sciadv.adz2240)

Science Advances
AAAS

Supplementary Materials for

The Human Organ Atlas

Claire L. Walsh *et al.*

Corresponding author: Claire L. Walsh, c.walsh.11@ucl.ac.ukf; Joseph Brunet, j.brunet@ucl.ac.uk; David Stansby, d.stansby@ucl.ac.uk; Peter D. Lee, peter.lee@ucl.ac.uk

Sci. Adv. **12**, eadz2240 (2026)
DOI: 10.1126/sciadv.adz2240

This PDF file includes:

Fig. S1

Fig. S1.

A) shows scanning speed (voxels / second) for scans since the start of the project in early 2021 for data in the Human Organ Atlas. Zoom datasets (blue points) are typically scanned at speeds between 2 x 10^7 and 8 x 10^7 voxels/s. Overview datasets (orange points) show an improvement in scanning speed over time due to technical developments, from around 2 x 10^6 voxels/s earlier in the project to similar speeds as zoom datasets for more recent datasets

B) shows scan time for a given physical volume at 2μm (blue line), 6μm (orange line), and 20μm (green line) voxel sizes, assuming a scan speed of 3 x 10^7 voxels/second. Typical scan times for large organs at 20μm voxel size are a few hours, with sub-hour scan times at 20μm voxel size for smaller organs.

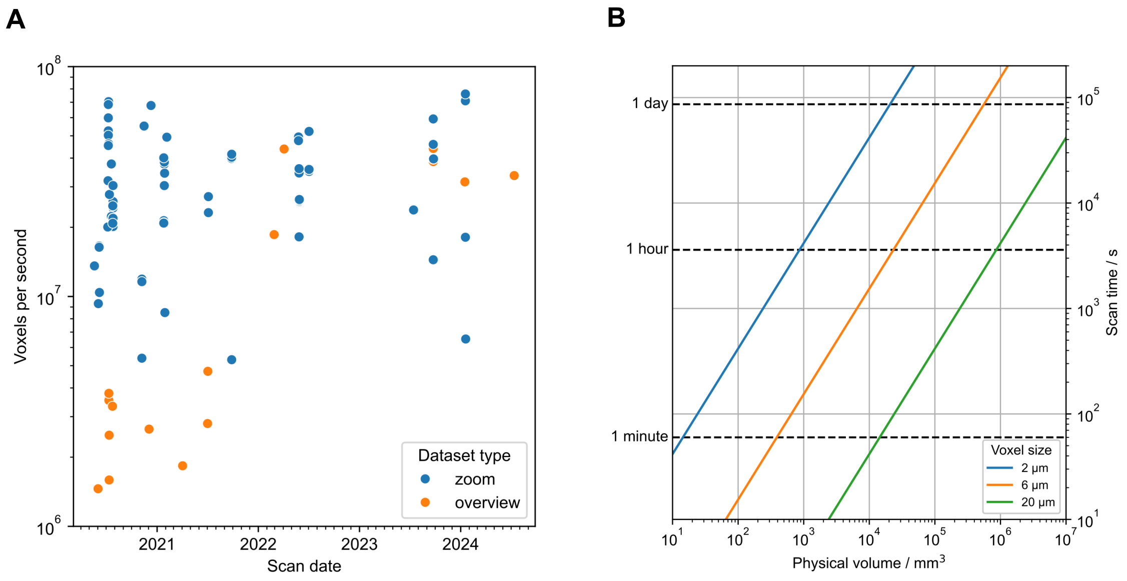

Supplement: Supplementary file 1 — Fig. S1 [file sciadv.adz2240_sm.pdf]
